# General Charities

**Published:** 1890-07-19

**Authors:** 


### GENERAL CHARITIES.

This Column is devoted to an account of work of charitable institutions other than Medical Charities. The Editor will be glad to receive reports or news of work at 140, Strand. *Philanthropy* should be plainly written on the envelope.

APPEALS for monetary help are coming in now thick and fast from every quarter, but the ones that for the moment claim our chief attention are those from the various societies who send poor children for a few weeks' holiday into the country. The summer is here, and hot August will soon be with us, and the very thought of a stuffy back street in London in that month (rubbish and decaying vegetables littered about), being often the only play-ground of our London children, makes the most callous of us feel what a boon these societies are.

Are we not always conjecturing on how much misery and expense might be saved the world at large by a little "prevention," and yet it is marvellously hard to carry out our philosophical decisions effectually—generally on account of that everyday fact—that funds are lacking.

There are several very large Fresh Air Societies and small private ones innumerable. The Children's Country Holiday Fund is hard at work doing its best to send away the ever-increasing number of little beings who need their help, no slight task when we realize that they sent away 20,772 children last summer. The expenditure was £13,178, and the parents paid £4,474. This parents' contribution shows us one or two things, for example, that the poor are really ready to make some sacrifice for the good of their children, and it shows us that the fund sees that real kindness lies in helping people to help themselves, not in sapping independence, but in backing up individual effort. Experience proves that feeling is no safe guide to charity, and the thirty-six local committees of the Children's Country Holiday Fund find that the instances of such extreme poverty when not the slightest contribution can be spared by the parents are tolerably rare, and the general rule of the Society is that parents must give what share of the expenses they can afford. This rule of course is like every other, subject to exceptions. In cases of extreme destitution free tickets are given at the discretion of the committee. If there is one thing especially which commends itself to wide-minded people it is to see a work carried out in an unsectarian manner, and children of every denomination are helped by this Society. And for the rest it must be remembered by those who will give what they can for the children, that excepting at the Central Office the 500 London workers are all voluntary, so that the money is not wasted in expenditure. Ten shillings secures a holiday for a fortnight for one child, and the physical strength alone which is gained from the glimpse of country is not the only benefit that accrues; is it not something to remember that the mind receives purer, "healthier stuff out of which to build its day-dreams," fresh impulses, wider ideas of life, and the knowledge for the first time that the unlawful pleasures in the London streets are not the only joys in life.

The fête in aid of the **METROPOLITAN AND CITY POLICE ORPHANAGE** went off with spirit on Thursday last. To benefit from this institution the child's father must have been a subscriber. Unfortunately there is not enough room for all applicants, so the orphanage pays compassionate allowance of 2s. 6d. per week for 500 children for whom there is no room.

The **CONVALESCENT DINNERS SOCIETY** closed its work on July 4th, and will begin again on October 3rd. All the workers of this society are voluntary. Last year's expenses were only £15, while 7,308 dinners were given away, at a cost of £199. Clergy and ministers of every denomination and other visitors to the poor may be honorary members, and may recommend deserving cases. The greatest care is taken that the recipients are in need, and have been either discharged from hospital or pronounced convalescent after illness, accident, or childbirth. Most of us know what a help fourteen days' good dinners must be, for there is a great difference between convalescence and health. Hon. Secretary, 114, Queen's Gate, S.W. Funds much needed.

**DR. BARNARDO** has left for Canada, in connection with his Homes for Destitute Children.

A "sandwich" man is supposed to be paid 2s. a day; this is what the hirers pay, but by the time it has filtered through agents, &c., the wretched man receives about 1s. Some time ago one of the evening papers gave an account of how a sandwich man lived on 7s. a-week, but he died shortly after from the effects of it. The **SALVATION ARMY** has opened a labour bureau for unskilled labour of every sort, and their board-men are to actually receive 2s. a day. This is good and useful, and we venture to suggest to the S. A. that this sort of work will do more to help fellow-men than all the brass bands in the world.

The **LONDON FEMALE PENITENTIARY AND GUARDIAN SOCIETY** at Stoke Newington states in its eighty-second report that with every endeavour the committee do not succeed in raising the funds sufficiently for the necessary expenditure. This society trains the girls for service in respectable situations, and gives them an outfit when they leave. The chief industry is laundry work, which is gladly taken, and well done at moderate prices, and the laundries are spotlessly clean, and Mrs. Walter, the lady superintendent, will send any information. We should like to see meat or fish on the diet list every day, for laundry work is hard and tiring, but no doubt this could be done if the public will come forward and support this old institution, where there are no bolts or bars, and where no inmate is detained against her will.

